# Changes in cardiovascular risk factors among children and young adults with type 1 diabetes during the COVID‐19 pandemic compared to previous years—Results from the German DPV registry

**DOI:** 10.1111/1753-0407.13340

**Published:** 2023-01-09

**Authors:** Alexander J. Eckert, Sabine Linke, Karl‐Otfried Schwab, Thekla von dem Berge, Eckhard Schönau, Ibrahim Duran, Axel Dost, Christine Joisten, Heike Bartelt, Katarina Braune, Joachim Rosenbauer, Reinhard W. Holl

**Affiliations:** ^1^ Institute of Epidemiology and Medical Biometry, ZIBMT University of Ulm Ulm Germany; ^2^ German Centre for Diabetes Research (DZD) Neuherberg Germany; ^3^ Katholisches Kinderkrankenhaus WILHELMSTIFT gGmbH Children's Hospital Hamburg Germany; ^4^ Faculty of Medicine, Centre for Pediatrics and Adolescent Medicine, Division of Pediatric Diabetology, Endocrinology and Lipidology, Medical Centre University of Freiburg Freiburg Germany; ^5^ Diabetes Centre for Children and Adolescents Children's Hospital Auf der Bult Hannover Germany; ^6^ Faculty of Medicine and University Hospital, Department of Pediatrics University of Cologne Cologne Germany; ^7^ Faculty of Medicine and University Hospital, Centre of Prevention and Rehabilitation Uni Reha, University of Cologne Cologne Germany; ^8^ Department of Pediatrics University Hospital Jena Jena Germany; ^9^ Department for Physical Activity in Public Health, Institute of Movement and Neurosciences German Sport University Cologne Cologne Germany; ^10^ Cologne Centre for Prevention in Childhood and Youth / Heart Centre Cologne University Hospital of Cologne Cologne Germany; ^11^ Department of Endocrinology and Diabetology University of Leipzig, Hospital for Children and Adolescents Leipzig Germany; ^12^ Institute of Medical Informatics Charité – Universitätsmedizin Berlin Berlin Germany; ^13^ Department of Pediatric Endocrinology and Diabetes Charité ‐ Universitätsmedizin Berlin Berlin Germany; ^14^ Berlin Institute of Health at Charité Berlin Germany; ^15^ German Diabetes Center, Institute for Biometrics and Epidemiology Leibniz Center for Diabetes Research at Heinrich Heine University Düsseldorf Germany

**Keywords:** blood pressure, cholesterol, coronavirus, lipid, physical activity, sports, 血压, 胆固醇, 冠状病毒, 血脂, 体力活动, 运动

## Abstract

**Background:**

The diverse stages of the COVID‐19 pandemic led to several social circumstances that influenced daily life and health behavior.

**Purpose:**

To evaluate changes in cardiovascular risk factors and physical activity among children and young adults with type 1 diabetes (T1D) during the COVID‐19 pandemic in Germany compared to previous years.

**Methods:**

A total of 32 785 individuals aged 6–21 years at baseline with T1D from the German diabetes patient follow‐up (DPV) registry contributed data on 101 484 person‐years between 2016 and 2021. The first treatment year of each individual within this period was considered as baseline. Based on trends from 2016 to 2019, we estimated differences in body mass index‐SD score (BMI‐SDS), blood pressure (BP‐SDS), and lipid levels (non‐high‐density lipoprotein [non‐HDL]) between observed and predicted estimates for the years 2020 and 2021 using linear regression analysis standardized for age, diabetes duration, sex, and migratory background. The proportion doing organized sports and smoking cigarettes was analyzed using multivariable logistic regression models.

**Results:**

BMI‐SDS increased constantly from 2016 to 2021 without a significant increase above expected values for 2020/2021. Systolic BP‐SDS (difference observed vs. expected with 95% confidence interval, 2020: 0.10 [0.07–0.14], 2021: 0.17 [0.14–0.20]) and non‐HDL (2020: 2.7 [1.3–4.1] mg/dl, 2021: 4.1 [2.7–5.5] mg/dl) were significantly increased (all *p* < .001) in both pandemic years. The proportion of subjects participating in organized sports was reduced from over 70% in prepandemic years to 35%–65% in diverse stages/waves of the COVID‐19 pandemic. The percentage smoking cigarettes did not change.

**Conclusions:**

We describe an increase in BP and atherogenic lipid levels coinciding with a reduction in physical activity but no acceleration of the prepandemic increases in BMI‐SDS among young people with T1D during the COVID‐19 pandemic.

## Graphical abstract

**Highlights**

- Cardiovascular risk factors worsened during COVID‐19 in young people with type 1 diabetes.
- Blood pressure and atherogenic lipid levels were higher in 2020/2021 than expected.
- No further boost in 2020 and 2021 of the preexisting increase in body mass index‐SD score.
- A reduction in organized sports but no change in smoking behavior was observed.

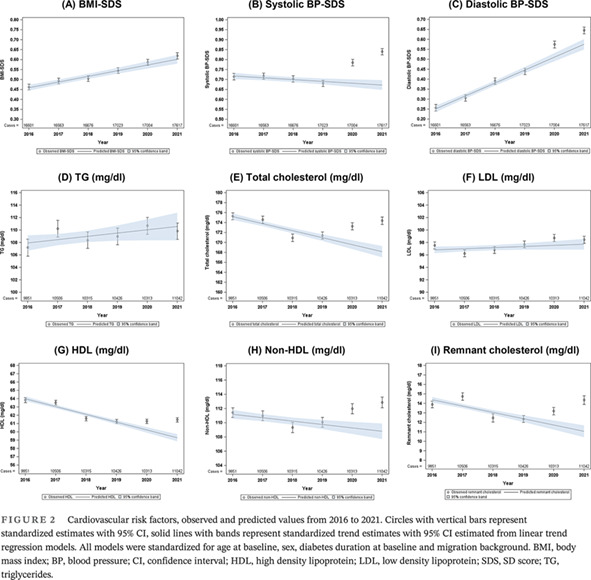

## 摘要

**目的:** 评估德国2019新型冠状病毒(COVID‐19)大流行期间，患有1型糖尿病(T1D)的青少年心血管风险因素和体力活动与往年相比的变化。

**方法:** 来自德国糖尿病患者随访登记(DPV)的32,785名6‐21岁基线T1D患者在2016年至2021年期间提供了101,484人‐年的数据。在这段时间内，每个人的第一个治疗年被视为基线。基于2016‐2019年的趋势，我们使用年龄、糖尿病病程、性别和迁移背景标准化的线性回归分析，估计了2020年和2021年的体重指数(BMI‐SDS)、血压(BP‐SDS)和血脂水平(非HDL)的观察值和预测值之间的差异。使用多变量逻辑回归模型分析有组织运动和吸烟的比例。

**结果:** 从2016‐2021年，BMI‐SDS持续上升，但没有显著高于2020/2021年的预期值。在两个大流行年份，收缩压BP‐SDS(观察到的与预期的差异，95% CI, 2020: 0.10[0.07‐0.14]， 2021: 0.17[0.14‐0.20])和非HDL (2020: 2.7 [1.3‐4.1] mg/dl, 2021: 4.1 [2.7‐5.5] mg/dl)显著升高(所有p<0.001)。参加有组织体育运动的受试者比例从大流行前年份的70%以上降至COVID‐19大流行不同阶段/波的35‐65%。吸烟的比例没有变化。

**结论:** 我们发现，在COVID‐19大流行期间，患有T1D的青少年的血压和致动脉粥样硬化性血脂水平升高，同时体力活动减少，但BMI‐SDS与疫情前相比并没有加速增加。

## 1 INTRODUCTION

Physical activity during childhood and adolescence is inversely associated with several cardiovascular risk factors. There is evidence that in children aged 4–18 years, systolic blood pressure (BP), waist circumference, triglycerides (TG), and high‐density lipoprotein (HDL) can benefit from moderate to vigorous physical activity.^1^ In children and adolescents with type 1 diabetes (T1D), physical activity can improve low‐density lipoprotein (LDL), TG, diastolic BP, and hemoglobin A1c (HbA1c).^2^ Smoking is another important lifestyle factor related to cardiovascular risk in children and adolescents, especially in those with diabetes.^3^, ^4^

The COVID‐19 pandemic caused by severe acute respiratory syndrome coronavirus 2 (SARS‐CoV‐2) reached Germany in February 2020, leading to a first nationwide lockdown from March to May and subsequent additional governmental health measures to contain the pandemic. The incidences of COVID‐19 fell during the summer months (June to September), but increased dramatically in a second (October 2020 to February 2021) and consecutive third wave (March to May 2021). Again, during summer 2021 the COVID‐19 incidence was relatively low and increased again since October 2021.^5^, ^6^

The fear of infection with COVID‐19 as well as the temporary closure of schools, sports clubs, and other public institutions led to isolation and lifestyle changes, especially a reduction of physical activity in children and adolescents worldwide, most pronounced especially during the first lockdown in 2020.^7^, ^8^, ^9^ According to a recent meta‐analysis, a reduction in physical activity during the COVID‐19 pandemic was observed in several countries.^10^ This could have led to long‐lasting health issues such as an increase in cardiovascular risk factors. However, in Germany, only organized sports were reduced during the first lockdown, together with an increase in sedentary time and the use of media during leisure time, but higher physical activity due to unorganized sports such as playing outdoors was observed in children.^11^ Further, there seemed to be remarkable differences regarding socioeconomic factors enabling children with better housing situations to engage in more habitual physical activity.^12^

Children and adolescents with T1D are particularly at risk for hypertension, dyslipidemia, increased periaortic fat thickness,^13^ and accelerated atherosclerosis.^14^ Many studies concentrated on glycemic control in children and adolescents with T1D during the COVID‐19‐related lockdown, reporting worse or stable HbA1c,^15^, ^16^ but data on cardiovascular risk, especially on BP and lipids during the pandemic, are still scarce.

The aim of this study was to evaluate changes in BP, serum lipids, physical activity, and smoking behavior in young individuals with T1D during the COVID‐19 pandemic in Germany compared to previous years, based on data from a German diabetes patient registry.

## 2 MATERIALS AND METHODS

### 2.1 Data collection

This analysis is based on data from the prospective, multicenter diabetes patient follow‐up registry (DPV, Diabetes‐Patienten‐Verlaufsdokumentation), which is a standardized electronic health record developed at the Institute of Epidemiology and Medical Biometry, Ulm University, Germany.^17^ The initiative and the analysis of anonymized data was approved by the Ethics Committee of Ulm University (approval number: 314/21) as well as by local review boards.

A total of 512 diabetes centers from Germany, Austria, Switzerland, and Luxembourg provided pseudonymized data on diabetes treatment and outcome to the registry until March 2022. Because of differences in societal responses among countries, only 300 German centers were included in this report, representing an estimated coverage of about 90% of children with diabetes in Germany.^18^ The transmitted data are checked for inconsistency or implausibility and reported back to the respective centers for correction, if necessary.

### 2.2 Participants and evaluation periods

**FIGURE 1:**
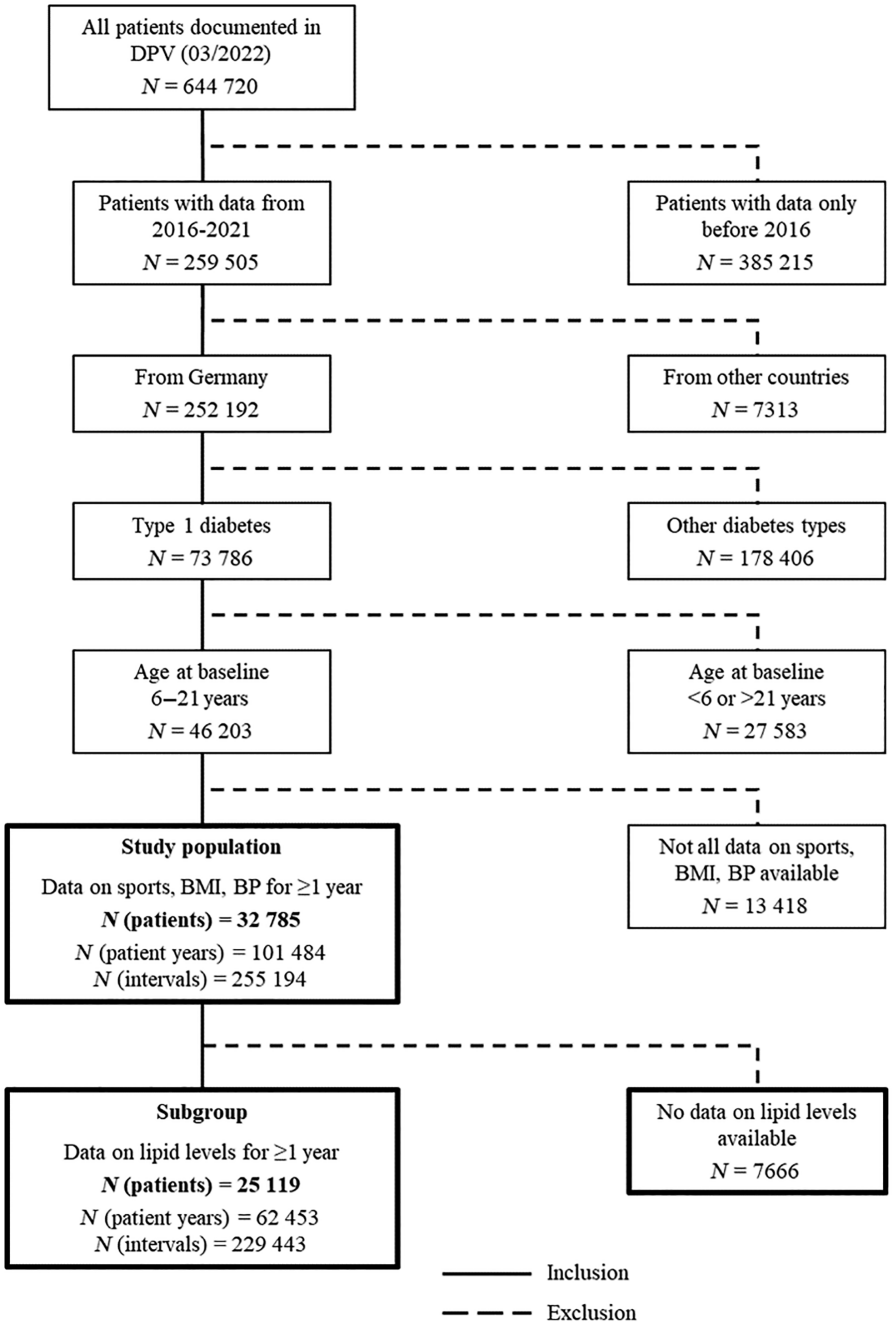
Inclusion and exclusion criteria of study population. Intervals = number of all observation intervals included in the analysis (1–24 per participant); lipid levels include total cholesterol, low‐density lipoprotein, high density lipoprotein (HDL), non‐HDL, remnant cholesterol, triglycerides. Baseline = first treatment year per patient from 2016–2021. BMI, body mass index (as SD score). BP, blood pressure (systolic and diastolic, as SD score). DPV, Diabetes‐Patienten‐Verlaufsdokumentation.

German individuals recorded in the DPV initiative were included if they had documented data at least in 1 year between 2016 and 2021 (Figure 1). The first year of each individual during this time frame was considered as baseline. The six treatment years were further divided into four periods according to the course of the COVID‐19 pandemic and associated policy measures in 2020 and 2021 in Germany to allow detailed analysis of lifestyle factors and treatment with antihypertensive or lipid‐lowering medication. Therefore, January and February were defined as period 1, before COVID‐19 in 2020. March to May was determined as period 2, representing the first wave of COVID‐19 and the first lockdown in 2020; June to September was defined as period 3 (summer) with lower COVID‐19 cases in both years; October to December was set as period 4, representing the increasing COVID‐19 incidences in Germany in both years.

Data were aggregated for each individual and year for analysis of the main outcomes body mass index (BMI), BP, and lipid values. Additionally, data were aggregated for each individual, year and the aforementioned period to analyze lifestyle factors. Therefore, all participants contributed data in 1–6 years and in 1–24 observation intervals (years*periods) to this analysis. Further inclusion criteria were a clinical diagnosis of T1D, age at baseline 6 ≤ 21 years and available data on systolic/diastolic BP, BMI, and organized sports in at least 1 year (Figure 1). If an individual had data on all outcomes for some, but not all years/observation intervals, observation intervals with missing data were omitted. A subgroup of individuals with available data on lipid values (total cholesterol [TC], TG, LDL, HDL) in at least 1 year was built for a separate analysis (Figure 1).

### 2.3 Patient data

For height, weight, BMI, and BP, SD scores (SDS) based on German reference values were used (anthropometry: AGA (Consortium of Obesity in Childhood and Adolescence as part of the German Obesity),^19^ blood pressure: KiGGS (German Health Interview and Examination Survey for Children and Adolescents).^20^ Lipid values were converted to mg/dl. Non‐HDL was calculated as TC minus HDL and remnant cholesterol (RC) as TC minus LDL and HDL. HbA1c values were standardized to the Diabetes Control and Complications Trial reference range of 4.05%–6.05% (20.7–42.6 mmol/mol) using the multiple of the mean transformation method to account for different laboratory methods.^21^ Physical activity was defined as organized/club sports (individual exercise at home was not included).^22^ Unfortunately, data on the sedentary time and the use of media was not available. Smoking was defined as current smoking of cigarettes (yes/no). Missing data were set as no smoking. Migratory background was defined as the patient or at least one of his/her parents born outside of Germany.

### 2.4 Statistical analysis

All statistical analyses were generated using SAS (Statistical Analysis Software, SAS Institute Inc., Cary, NC, USA) Version 9.4, Built M7, on a Windows Server 2016 mainframe. Descriptive statistics were performed for all patients at baseline. The results are shown as median with quartiles for continuous variables and as proportions for binary variables.

The proportions of individuals doing organized sports, smoking cigarettes, with prescribed antihypertensive or lipid‐lowering medication as well as the proportion with overweight or obesity (BMI‐SDS ≥ 90th percentile, representing an SDS value ≥ 1.28) and with obesity (BMI‐SDS ≥ 97th percentile, representing an SDS value ≥ 1.88) by year and period were analyzed using multivariable logistic regression models adjusted for age groups (6–11, >11–16, >16–21 years at baseline), sex, diabetes duration groups (≤2, >2–6, >6 years at baseline), and migratory background. To consider repeated measurements of individuals that contributed more than one observation interval, a random effect (random residual for each subject) for the period*year‐interaction was implemented with a first‐order autoregressive covariance structure and an optimization technique of Newton–Raphson with ridging.

To analyze deviations of the respective outcome during the COVID‐19 pandemic (years 2020 and 2021) compared to predicted levels based on the prepandemic years (2016–2019), we first applied multivariable linear trend regression models from 2016 to 2019 for BMI, systolic and diastolic BP, and lipid levels (TC, LDL, HDL, non‐HDL, RC, and TG). Independent variables were treatment year (continuous), age groups, and diabetes duration groups at baseline, sex, and migratory background. Again, repeated measurements of individuals with data from more than 1 year were considered with a random effect of each subject for treatment years. Based on these trend models, we predicted the outcomes for 2016 to 2021, standardized for the aforementioned confounders. Second, a linear regression model with the same standardization, but the year as a categorial term was used to estimate the observed outcome levels for each year (2016–2021). Third, a linear regression model that included a binary variable indicating observed and predicted data as independent variables was applied to compare the observed with the predicted outcome levels. As a measure of deviation, the difference between these observed and predicted outcome levels was estimated as the standardized difference with the corresponding 95% confidence intervals (CI) and two‐sided *p* values from the Wald test. Sensitivity analysis was conducted with further adjustment for BMI‐SDS groups (</≥ 90th percentile) and the proportion with antihypertensive medication (for systolic and diastolic BP) or the proportion with lipid‐lowering medication (for lipid levels). We additionally analyzed non‐HDL only in individuals with good glycemic control (HbA1c <7.5%). Two‐sided *p* < .05 indicated a significant difference.

## 3 RESULTS

### 3.1 Study population

Among all individuals registered in DPV, 32 785 were aged 6 ≤ 21 years at baseline, had a diagnosis of T1D, were from Germany, and fulfilled the inclusion criteria. These individuals provided a total of 101 484 patient‐years. Figure 1 gives an overview on the inclusion criteria and the number of patients and observation intervals.

Included individuals had a median age at baseline of 12.8 [9.5; 15.5] years with 53% being male and 25% having a migratory background, and 76% were physically active. Table 1 displays further characteristics at baseline.

**TABLE 1:** Patient characteristics at baseline, *N* = 32 785.

Characteristic	Missing (*N*)	%	Median	Q1	Q3
Sex (% male)		52.8			
Age (years)			12.8	9.5	15.5
Diabetes duration (years)			2.9	0.8	6.2
Height‐SDS			0.1	−0.6	0.7
Weight‐SDS			0.4	−0.2	1.1
BMI‐SDS			0.4	−0.2	1.1
Doing organized sports		76.1			
Smoking cigarettes		3.2			
HbA1c (%)	280		7.4	6.6	8.2
HbA1c (mmol/mol)	280		57	49	66
Systolic BP (mmHg)			115	107	124
Systolic BP (SDS)			0.7	−0.0	1.4
Diastolic BP (mmHg)			68	62	74
Diastolic BP (SDS)			0.3	−0.4	1.0
Hypertension		3.8			
Antihypertensive medication		1.4			
Total cholesterol (mg/dl)	14 206		170	151	192
LDL (mg/dl)	15 181		94	77	113
HDL (mg/dl	15 116		62	52	72
Non‐HDL (mg/dl)	15 439		106	89	127
Remnant cholesterol (mg/dl)^a^	15 664		12	6	19
Triglycerides (mg/dl)	14 915		89	62	129
Fasting triglycerides (mg/dl)	29 506		76	54	115
Proportion TG fasting^b^	14 915	18.4			
Dyslipidemia	13 834	24.3			
Non‐HDL > 140 mg/dl	15 439	15.4			
Lipid‐lowering medication		0.4			

Abbreviations: BMI, body‐mass index; BP, blood pressure; HbA1c, Hemoglobin A1c; HDL, high density lipoprotein; LDL, low density lipoprotein; Q1, lower quartile; Q3, upper quartile; SDS, SD score, reference AGA (height, weight, BMI) or KiGGS (systolic/diastolic blood pressure); TG, triglycerides.

^a^ Remnant cholesterol = Total cholesterol − (LDL + HDL).

^b^ Proportion of TG measurements that were performed fasting.

### 3.2 Cardiovascular risk factors by treatment year

**FIGURE 2:**
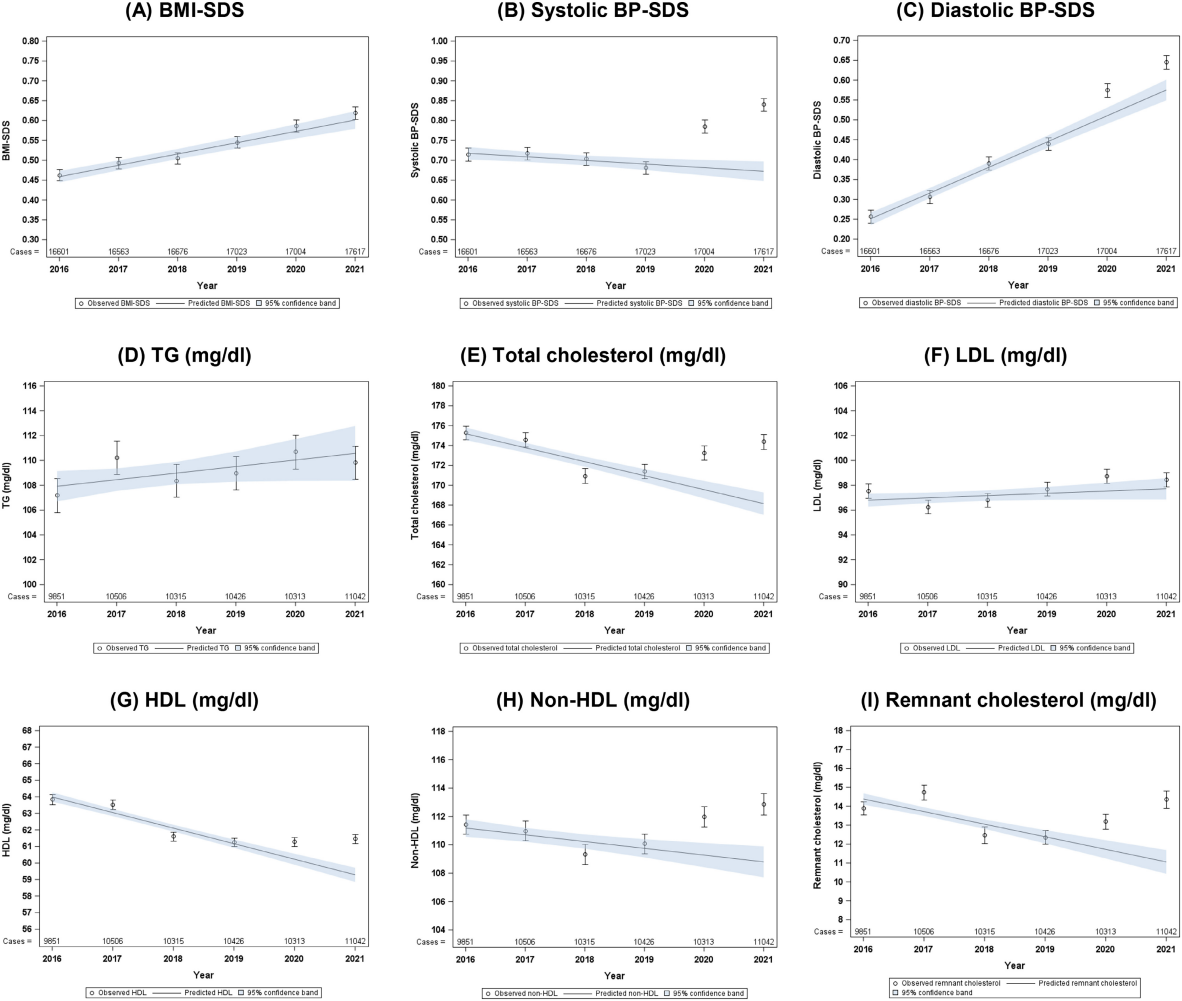
Cardiovascular risk factors, observed and predicted values from 2016 to 2021. Circles with vertical bars represent standardized estimates with 95% CI, solid lines with bands represent standardized trend estimates with 95% CI estimated from linear trend regression models. All models were standardized for age at baseline, sex, diabetes duration at baseline and migration background. BMI, body mass index; BP, blood pressure; CI, confidence interval; HDL, high density lipoprotein; LDL, low density lipoprotein; SDS, SD score; TG, triglycerides.

Yearly analysis of cardiovascular risk factors showed an ongoing trend in BMI‐SDS increase from 2016 to 2021, but no further worsening during the COVID‐19 pandemic in 2020 or 2021 (Figure 2A). Nevertheless, systolic BP was higher in 2020 and in 2021 than expected (standardized difference with 95% CI, observed vs. expected: 2020: 0.10 [0.07; 0.14], *p* = .001 and 2021: 0.17 [0.14; 0.20, *p* = .001]). This implies an increase of 15% (2020) and 25% (2021) compared to the expected systolic BP. Similar results could be observed for diastolic BP with an increase of 13%/12% compared to expected diastolic BP in 2020 and 2021, despite a previously increasing trend since 2016 (Figure 2B,C and Table 2). This increase in BP was still significant, even with further adjustment for BMI‐SDS and for antihypertensive treatment (Figure S1).

**TABLE 2:** Cardiovascular risk factors, trend 2016–2019 and standardized difference between observed and expected values for 2020 and 2021.

Outcome	Trend 2016–2019 (change per year)	Difference observed vs. expected, 2020	Difference observed vs. expected, 2021
BMI‐SDS	0.03 (0.02; 0.03), *p* < .001	0.01 (−0.02; 0.04), *p* = .308	0.02 (−0.01; 0.05), *p* = .308
Systolic BP‐SDS	−0.009 (−0.015; −0.003), *p* = .005	0.10 (0.07; 0.14), *p* < .001	0.17 (0.14; 0.20), *p* < .001
Diastolic BP‐SDS	0.06 (0.06; 0.07), *p* < .001	0.06 (0.03; 0.10), *p* < .001	0.07 (0.04; 0.10), *p* < .001
TG (mg/dl)	0.5 (−0.1; 1.1), *p* = 0.075	0.7 (−2.1; 3.4), *p* = .999	−0.7 (−3.4; 2.0), *p* = .999
Total cholesterol (mg/dl)	−1.4 (−1.7; −1.1), *p* < .001	3.7 (2.2; 5.2), *p* < 0.001	6.2 (4.8; 7.7), *p* < .001
LDL (mg/dl)	−0.18 (−0.04; −0.40), *p* = .102	1.2 (0.1; 2.3), *p* = .028	0.7 (−0.3; 1.8), *p* = .121
HDL (mg/dl)	−0.9 (−1.1; −0.8), *p* < .001	1.1 (0.5; 1.6), *p* < .001	2.2 (1.7; 2.7), *p* < .001
Non‐HDL (mg/dl)	−0.5 (−0.8; −0.2), *p* < .001	2.7 (1.3; 4.1), *p* < .001	4.1 (2.7; 5.5), *p* < .001
Remnant cholesterol (mg/dl)	−0.7 (−0.8; −0.5), *p* < .001	1.5 (0.7; 2.3), *p* < .001	3.3 (2.5; 4.1), *p* < .001

*Note*: The trend 2016–2019 describes the estimated change per year in the respective unit (SDS or mg/dl), standardized for age groups at baseline, sex, diabetes duration at baseline, and migration background. Estimated differences between observed and expected (based on trend 2016–2019) values are presented in the same unit and standardized for the same variables.

Abbreviations: BMI, body‐mass index; BP, blood pressure, HDL, high density lipoprotein; LDL, low density lipoprotein; SDS, SD score; TG, triglycerides.

For lipid levels, we detected higher observed vs. expected values for total cholesterol, non‐HDL (difference observed vs. expected in 2020: 2.7 [1.3; 4.1] mg/dl, *p* < .001 and in 2021: 4.1 [2.7; 5.5] mg/dl, *p* < .001), and RC (Figure 2E/H/I and Table 2) but not for TG or LDL (Figure 2D,F and Table 2). HDL was also significantly increased in both years (Figure 2G and Table 2). Again, these results persisted with further adjustment for BMI‐SDS and lipid‐lowering medication (Figure S1). An increase in non‐HDL was also detected in a subgroup with well‐controlled HbA1c (<7.5%, Figure S1H). Figure 2 and Table 2 show the trend from 2016 to 2019 as well as the standardized differences between observed and expected values for all outcomes.

### 3.3 Results by year and period

**FIGURE 3:**
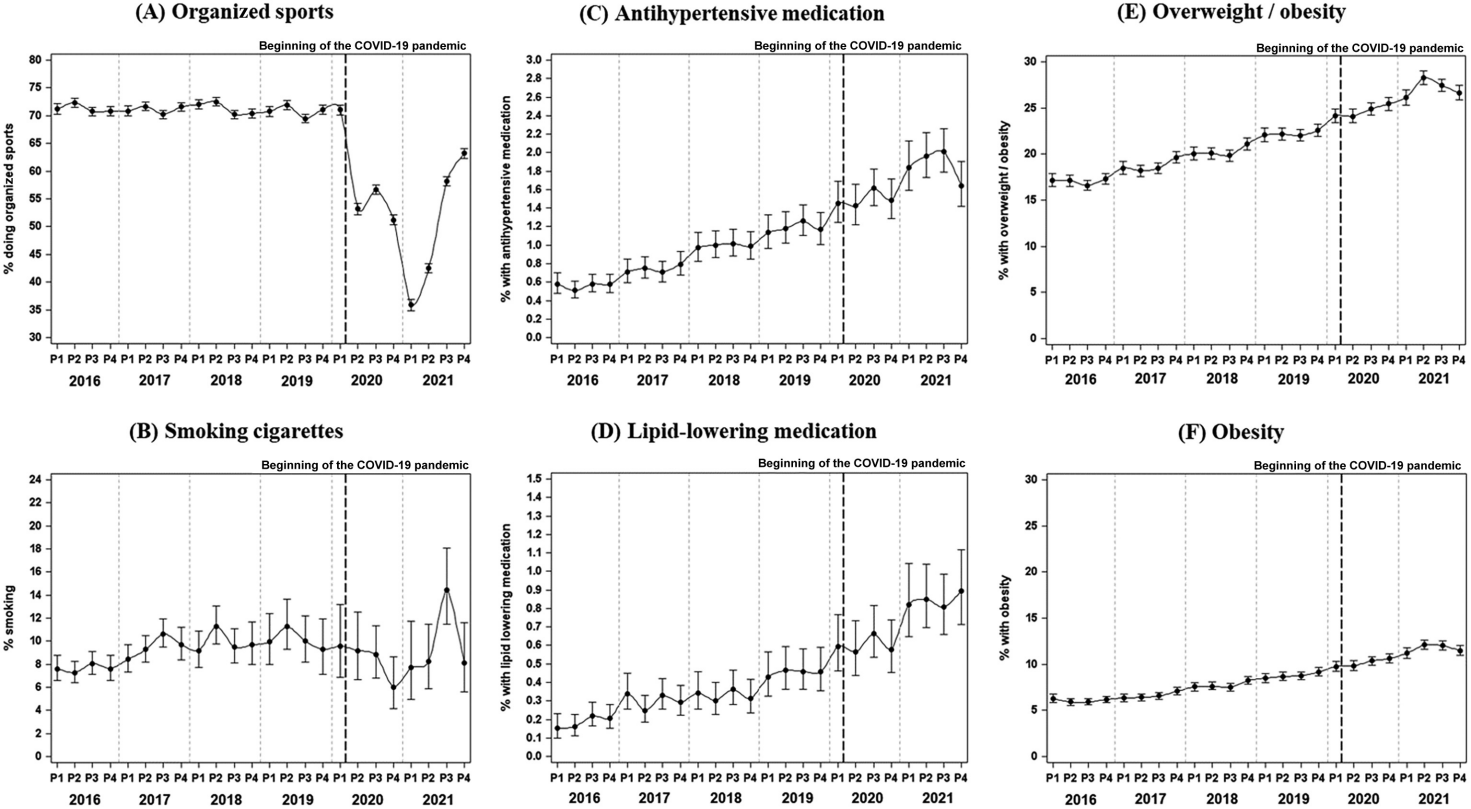
Proportion (%) of lifestyle factors and prescribed medication by year and period. Shown are results from multivariable regression models, adjusted for age, sex, diabetes duration, and migratory background, with random effect for repeated measurements. Outcomes are (A) organized sports, (B) smoking cigarettes, (C) with prescribed antihypertensive medication, (D) with prescribed lipid‐lowering medication, with overweight or obesity defined as BMI‐SDS ≥90th percentile (E), with obesity defined as BMI‐SDS ≥97th percentile (F). Missing values on smoking were assumed as no smoking. Excluding these missing data provided similar results, data not shown). BMI‐SDS, body mass index‐SD score.

The analysis by year and period revealed a significant (all *p* < .001) decrease in the proportion of children and adolescents doing organized sports at least once per week starting in March 2020, from constantly over 70% in the prepandemic observation periods to 35%–65% during the diverse periods of the COVID‐19 pandemic in 2020 and 2021 (Figure 3A). The proportion of children and young adults smoking cigarettes stayed relatively stable since 2016; only during summer 2021, an increase was observed (Figure 3B). The percentage with prescribed antihypertensive and lipid‐lowering medication (Figures 3C,D) as well as the proportions of individuals with overweight and with obesity (Figures 3E,F) constantly increased from 2016 to 2021 without a clear acceleration in the two pandemic years.

## 4 DISCUSSION

We found significantly elevated systolic and diastolic blood pressure and higher atherogenic lipid levels during the COVID‐19 pandemic in Germany compared to the expected values based on trend analyses from the years 2016–2019. This increase coincided with a reduction in the proportion of children and young adults with T1D doing organized sports, but no remarkable differences in the proportion smoking cigarettes and no acceleration of the prepandemic increase in BMI‐SDS.

BMI‐SDS rose constantly from 2016 to 2021, but there was no significantly higher increase in 2020 or 2021 than expected from the previous years. Therefore, no additional deterioration due to the lockdown periods and the pandemic itself on BMI‐SDS can be postulated by now. A study on children with T1D from India found no worsening in BMI *z* scores as well,^23^ whereas another study found increased body weight and BMI in pediatric T1D already during the first lockdown.^15^ However, these studies did not account for the preexisting trend and only compared BMI at a certain point of time before the pandemic and during the lockdown. This approach seems to be problematic as we discovered an increase in BMI‐SDS in young individuals with diabetes that occurred even before the beginning of COVID‐19. A Korean study group analyzed this BMI‐SDS trend over 15 years in children (with no regard to diabetes) and detected a constant increase in BMI‐SDS over this time span but the increase from 2019 to 2020 was smaller than the prepandemic increases per year^24^ which supports our results on a preexisting trend. Another limitation in the evaluation of BMI prepandemic and during the pandemic that must be kept in mind is the definition of BMI itself. Because BMI is only the ratio of body weight and squared body height, there could have been body fat independent changes, for example, lower muscle mass due to the decrease in physical activity, that might have limited the increase in body weight and therefore BMI during the COVID‐19 period, which does not exclude a worse body fat percentage or composition.

Higher BP (systolic and diastolic) in 2020 and 2021 compared to previous years goes parallel with the decrease in the proportion of individuals being physically active during the COVID‐19 pandemic so far. This is not surprising because physical activity can improve BP within hours^25^ as well as reduce hypertension in children with obesity.^26^ Therefore, inactivity might lead to higher BP in the long term. It is even suggested that the positive effects of physical activity on BP can be negated with excessive sedentary time.^27^ Unfortunately, there are still only few studies describing the association of the COVID‐19 pandemic with BP in children. A Chinese work group found elevated BP in children during the COVID‐19 quarantine,^28^ but studies on children with diabetes are scarce. It must be mentioned that other factors are likely to contribute to this increase in BP during the pandemic, such as stress, anxiety, or other psychological effects, as well as dietary habits that might have changed at least during the lockdown periods.

As TC, HDL, and non‐HDL were significantly higher in 2020 and 2021 than expected from the previous trend since 2016, one might assume that the detrimental (non‐HDL) and beneficial (HDL) effects could negate each other. However, it is discussed in the literature that an increase in non‐HDL as marker of the combined atherogenic lipids might be most important, especially in people with diabetes.^29^, ^30^ It is further suspected that remnant cholesterol might be another important marker for cardiovascular risk independently from traditional risk factors such as LDL^31^ and that HDL might have a U‐shaped – rather than a linear – association with cardiovascular risk.^32^ This is confirmed by a recent cohort study from the United Kingdom that identified very high HDL levels being associated with mortality in people with coronary artery disease.^33^ We, therefore, assume that the elevated levels of non‐HDL and RC could lead to a more atherogenic lipid profile during the COVID‐19 pandemic, despite the potentially beneficial effect of increasing HDL. This effect seems to be independent of weight status, as neither BMI‐SDS itself was higher than expected in 2020/2021 nor adjustment for BMI‐SDS changed these results. This would be in line with another observational study that found higher lipid values, but no significant changes in BMI *z*‐scores in young people with T1D aged 2–21 years.^34^ The shown development of hypertension and dyslipidemia depicts the need for early detection and adequate treatment of these cardiovascular risk factors in young people with T1D, because it is known that the management, especially of dyslipidemia, is suboptimal in adults with type 1 and type 2 diabetes^35^, ^36^ The increase of atherogenic lipid levels, namely non‐HDL, was although to a lower extent than in the whole study cohort also present in well controlled individuals with HbA1c <7.5% suggesting that this development does not only affect children and adolescents with worse glycemic control.

Owing to the closure of schools and sports clubs, the proportion of children and young adults with T1D doing organized sports decreased from over 70% in previous years to 35% at the beginning of 2021, recovering to about 65% at the end of 2021. Several previous studies have reported a worrying decline in physical activity in children and adolescents with and without diabetes.^8^, ^37^, ^38^ In regard to a recent meta‐analysis we have to assume that not only the percentage of individuals doing organized sports but also the amount of physical activity, especially with high intensity, was lower.^10^ As in the DPV registry, only club sports are accurately documented, children might have played outdoors to a higher extent, adolescents and young adults could have performed at‐home exercises or increased their habitual physical activity as suggested by a study on children during the first lockdown in Germany.^39^ However, mostly children with good socioeconomic status had the opportunity to compensate the loss of organized sports by being habitually active.^12^ It is known that during summer 2020, some sports facilities in European countries reopened occasionally, but most of them closed again in winter 2020/2021 resulting in an even higher decline in physical activity.^40^ This is in line with our findings on a short recovery in the proportion of doing organized sports from June to September 2020, followed by the lowest proportion in January and February 2021.

Smoking behavior did not reveal remarkable differences during the COVID‐19 pandemic compared to previous years. Other studies found quite varying lifestyle changes during the first wave of COVID‐19.^41^, ^42^ It might be individually different how to respond to a challenging situation with fear of infection and reduced peer interaction due to social distancing but also the possibility to structure one's life in a healthier way, for example, because of more control over smoking behavior of the children and better dietary habits because the parents worked partly at home and therefore had more time to provide healthier food.

The strength of this study was that >30 000 children and young adults with T1D, providing data on over 100 000 patient years between 2016 and 2021, could be analyzed, and therefore, reliable results were generated on the change in markers for cardiovascular risk, physical activity and lifestyle factors during the COVID‐19 pandemic compared to previous years. It is an important approach to take the preexisting trends of these risk factors into account to minimize error‐prone interpretations that could emerge when only a single time point before the pandemic is compared with a second time point within the COVID‐19 pandemic. Nevertheless, there are some limitations: In this diabetes patient registry, only data on organized sports were accurately documented. It is, therefore, possible, that some individuals conducted exercises at home as a reaction to the closure of sport clubs. For this reason, we confined ourselves to analyzing the proportion of children and young adults reporting any physical activity by period as documented in the DPV data set. Unfortunately, no data on sedentary time and use of media were available. Further, some patients might not (or less frequently) have seen their diabetologists during the pandemic, which might have led to bias in the population before and within the pandemic. We tried to consider this by analyzing aggregated data per year for the main outcomes, as it is likely that children and young adults with diabetes had at least one visit per year, even during the two pandemic years. It must be stated that our data cannot provide causality; we can only speculate about underlying reasons.

Taken together, we assume that there is a worsening in BP and lipid profile present over a period of 2 years by now in this already vulnerable and young population with T1D. This deterioration in cardiovascular risk factors seems to be independent of the ongoing increase in BMI‐SDS. Cigarette smoking is assumingly not strongly associated with the increase in BP and lipid values during the COVID‐19 pandemic. The reduced physical activity together with increased sedentary time, dietary habits, and psychological as well as socioeconomic aspects might be more important for this development. Although organized sports seemed to go back to near normal availability by the end of 2021, the differences between observed and predicted BP and non‐HDL were even higher in 2021 than in 2020. Therefore, it will be important to follow these individuals in future studies to investigate how cardiovascular outcomes develop during the ongoing pandemic.

## AUTHOR CONTRIBUTIONS

Alexander J. Eckert analyzed the data and wrote the first draft of the paper. Reinhard W. Holl conceived and coordinated the study and reviewed the article critically. Joachim Rosenbauer helped with statistical analysis and reviewed the article critically. The authors Sabine Linke, Karl‐Otfried Schwab, Thekla von dem Berge, Eckhard Schönau, Ibrahim Duran, Axel Dost, Christine Joisten, Heike Bartelt, and Katarina Braune participated in the DPV initiative by providing data and reviewed the article critically.

## FUNDING INFORMATION

This study was supported through the German Federal Ministry for Education and Research within the German Centre for Diabetes Research (DZD, 82DZD14E03). Further financial support was received by the German Robert Koch Institute (RKI), the German Diabetes Association (DDG) and by the German Diabetes Foundation (DDS, FP‐0446‐2022). Sponsors were not involved in data acquisition or analysis.

## CONFLICT OF INTEREST

The authors declare no conflict of interest.

## Supporting information


**Figure S1.** Cardiovascular risk factors, observed and predicted values from 2016 to 2021—sensitivity analysis.
Circles with vertical bars represent standardized estimates with 95% confidence interval (CI), solid lines with bands represent standardized trend estimates with 95% CI estimated from linear trend regression models. All models were standardized for age at baseline, sex, diabetes duration at baseline, migration background and additionally for body mass index‐SD score (BMI‐SDS) (</ ≥90th percentile) and prescribed medicine. Well controlled implies individuals with hemoglobin A1c (HbA1c) <7.5%.
