# Supplementary figures and images for: Changes in cardiovascular risk factors among children and young adults with type 1 diabetes during the COVID‐19 pandemic compared to previous years—Results from the German DPV registry
Source: J Diabetes. 2023 Jan 9;15(1):15–26. doi: 10.1111/1753-0407.13340 (PMC9870744; doi:10.1111/1753-0407.13340)

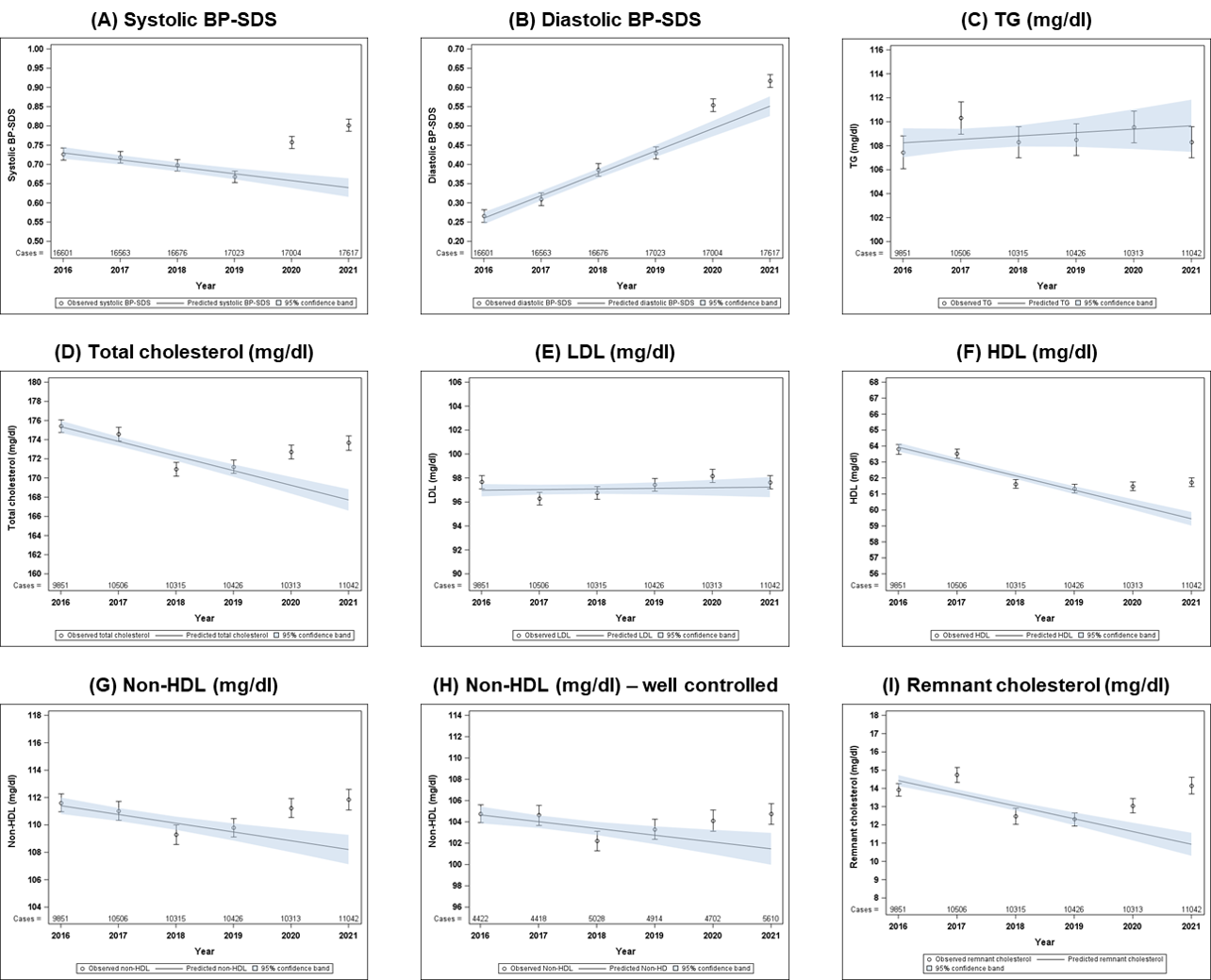

Supplement: Supplementary file 1 — Figure S1. Cardiovascular risk factors, observed and predicted values from 2016 to 2021—sensitivity analysis. Circles with vertical bars represent standardized estimates with 95% confidence interval (CI), solid lines with bands represent standardized trend estimates with 95% CI estimated from linear trend regression models. All models were standardized for age at baseline, sex, diabetes duration at baseline, migration background and additionally for body mass index‐SD score (BMI‐SDS) (</ ≥90th percentile) and prescribed medicine. Well controlled implies individuals with hemoglobin A1c (HbA1c) <7.5%. [file JDB-15-15-s001.tif]
